# How could the topic patient safety be embedded in the curriculum? A recommendation by the Committee for Patient Safety and Error Management of the GMA

**DOI:** 10.3205/zma001162

**Published:** 2018-02-15

**Authors:** Jan Kiesewetter, Sabine Drossard, Rainer Gaupp, Heiko Baschnegger, Isabel Kiesewetter, Susanne Hoffmann

**Affiliations:** 1 Klinikum der LMU München, Institut für Didaktik und Ausbildungsforschung in der Medizin, Munich, Germany; 2 Munich Municipal Hospital Group, Schwabing Hospital, Paediatric Surgery Department, Munich, Germany; 3 Albert-Ludwigs University of Freiburg, Medical psychology and sociology, Freiburg, Germany; 4 Hospital of the Ludwig-Maximilians-University Munich, Institute for Emergency Medicine and Management in Medicine (INM), Munich, Germany; 5 Hospital of the Ludwig-Maximilians-University Munich, Department of Anaesthesiology, Munich, Germany; 6 University Hospital Bonn, Institute for Patient Safety, Bonn, Germany

**Keywords:** patient safety, medical education, curriculum development, learning objectives, competences

## Abstract

The topic of patient safety is of fundamental interest for the health care sector. In view of the realisation of the National Competence-Based Learning Objectives Catalogue for Undergraduate Medical Education (NKLM) this topic now has to be prepared for medical education. For a disciplinary and content-related orientation the GMA Committee developed the Learning Objectives Catalogue Patient Safety for Undergraduate Medical Education (GMA-LZK). To ensure an optimal implementation of the GMA-LZK we recommend a longitudinal embedding into the existing curriculum. This position paper supports the implementation of the GMA-LZK and is aimed at everyone who wants to establish teaching courses on the topic patient safety and embed them in the curriculum. In light of this, we will initially describe the key features for a structured analysis of the current situation. Based on three best-practice-examples, as seen in the faculties of Freiburg, Bonn and Munich, different approaches to the implementation of the GMA-LZK will be illustrated. Lastly, we will outline the methodical requirements regarding the curriculum development as well as the disciplinary and methodical competences that the lecturers will have to hold or develop to fulfil the requirements.

## Zusammenfassung

Das Thema Patientensicherheit steht im Zentrum des Interesses des Gesundheitswesens. Im Hinblick auf die Umsetzung des Nationalen Kompetenzbasierten Lernzielkataloges für das Medizinstudium (NKLM) muss das Thema nun für die medizinische Lehre vorbereitet werden. Zur fachlich-inhaltlichen Orientierung wurde vom GMA-Ausschuss der Lernzielkatalog Patientensicherheit für das Medizinstudium (GMA-LZK) entwickelt. Für eine optimale Umsetzung des GMA-LZK wird eine longitudinale Einbettung in das bestehende Curriculum empfohlen. Das vorliegende Positionspapier soll als Hilfestellung zur Implementierung des GMA-LZK dienen und richtet sich an alle Personen, die Lehre zum Thema Patientensicherheit etablieren und im Curriculum verankern möchten. In diesem Zusammenhang werden zunächst Eckpunkte für eine strukturierte Standortanalyse beschrieben. Anhand dreier Best-Practice-Beispiele der Fakultäten Freiburg, Bonn und München wird aufgezeigt, welche unterschiedlichen Herangehensweisen zur Implementierung des GMA-LZK bereits genutzt werden. Abschließend wird dargestellt, welche methodischen Anforderungen sich für die Curriculumsentwicklung ergeben und welche fachlichen und methodischen Kompetenzen Lehrende mitbringen bzw. entwickeln müssen, um den Anforderungen gerecht zu werden.

## Introduction

Within the scope of the “Masterplan Medizinstudium 2020” (Master Plan Undergraduate Medical Education 2020) medical education once again faces far-reaching changes, that challenges all faculties but offers great opportunities at the same time. The significance of patient safety is being acknowledged in medical education like never before. Therefore, it is now being considered and integrated as teaching and educational content when discussing curricular reforms, currently above all in the course of the implementation of the National Competence-Based Learning Objectives Catalogue for Undergraduate Medical Education (NKLM). 

The topic of patient safety neither belongs to a single clinical or theoretical subject nor is institutionally directly represented by a specialist discipline, but it impacts on all areas of medicine. Therefore, the question arises of how to still achieve a successful and structured implementation of patient safety teaching contents. At the same time, it should be taken into account that the topic of patient safety affects all professions, levels of hierarchy and disciplines on a multiprofessional and interdisciplinary level and currently already influences and for most parts has always, at least implicitly, influenced the curriculum in different areas. 

At present the goal of integrating the topic of patient safety into the curricula in a structured and comprehensive manner seems feasible – knowing perfectly well that the didactic and content design of the teaching units on patient safety has not yet been completed. The GMA Committee for Patient Safety and Error Management follows this process and can already witness efforts being made by many faculties to integrate courses on the topic patient safety in undergraduate medical education. 

For didactic and conceptual guidance the GMA Committee developed the Learning Objectives Catalogue Patient Safety for Undergraduate Medical Education (GMA-LZK) [1]. This learning objectives catalogue explicitly addresses the undergraduate medical education at German universities; medical training programmes for doctors are not factored in. The GMA-LZK consists of a total of 38 learning objectives that are marked accordingly as cognitive objectives, attitude objectives and/or as skills. All learning objectives of the GMA-LZK are referenced to the NKLM. They are structured in three consecutive thematic blocks encouraging a longitudinal implementation. Contrary to other learning objectives catalogues, the GMA-LZK contains comparatively few but practical and tangible learning objectives that represent the basic knowledge and skills of future doctors in the field of patient safety [1].

## Objectives and outline

The starting points and the parameters for a curriculum development vary greatly at the medical faculties. Therefore, we expect different variations of implementing the topic of patient safety to be necessary and possible. However, there are fundamental aspects and questions at every faculty that have to be addressed and/or resolved even before efforts for the implementation in general and regarding specific contents of patient safety are being made. 

Consequently, this position paper addresses everyone that wants to establish teaching courses on patient safety either in regard to patient safety within the framework of the GMA-LZK or beyond and embed them in the curriculum. The Committee for Patient Safety and Error Management composed this position paper over the course of three workshops in Munich and Bern throughout the years 2016 and 2017. By the last page the reader should

- know that patient safety can be integrated into the undergraduate medical education by various means and
- be able to make assessments as to how the curriculum development at the own faculty can be moved forward constructively and within resource limits.

## General information on the implementation of the GMA-LZK

**Figure 1:**
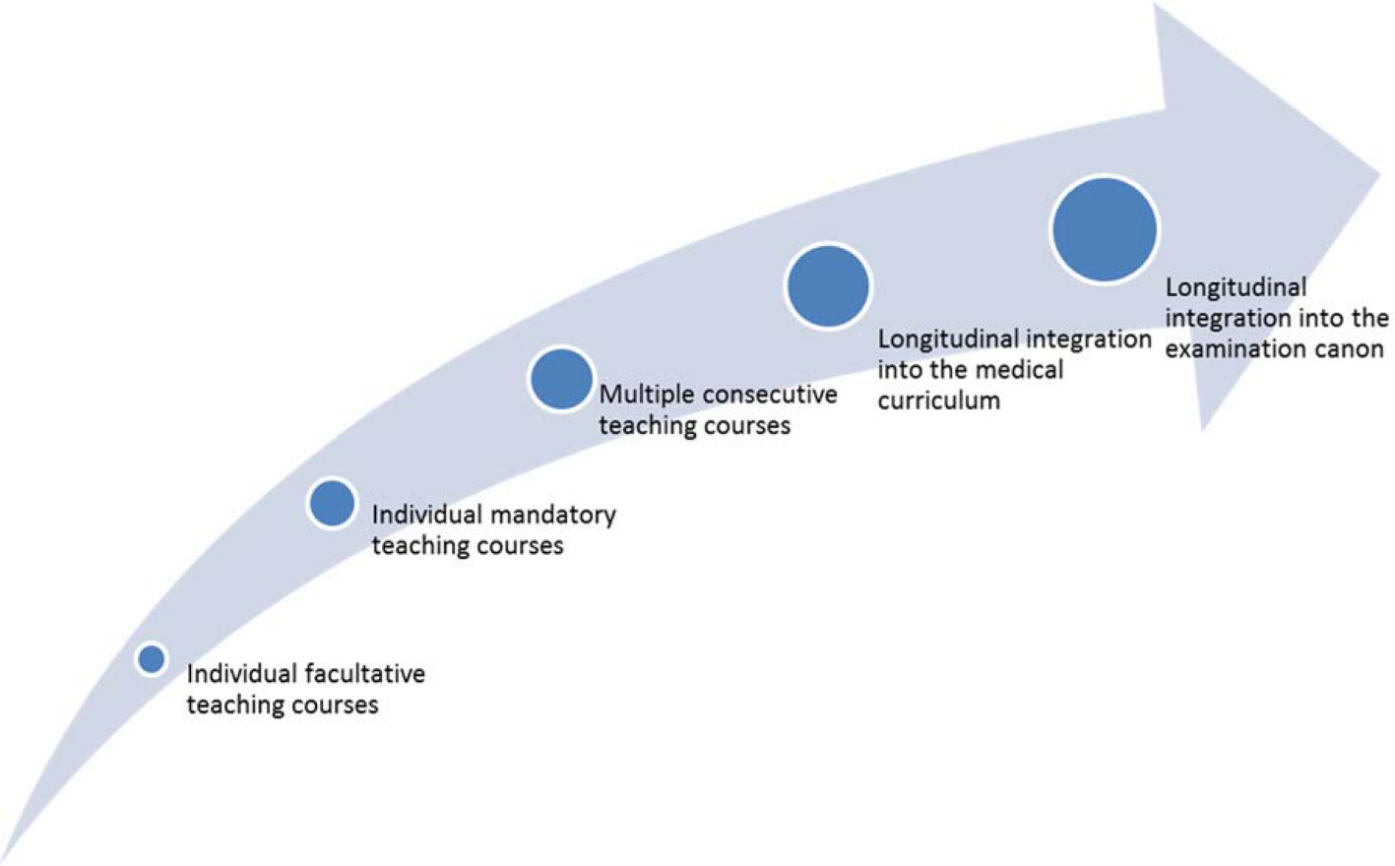
Longitudinal curriculum development

First we discuss some general information on and recommendations for the implementation of the GMA-LZK. In principle, the actions taken to develop a curriculum should be based on the iterative cycle by Kern [2]. We recommend a longitudinal embedding of the Learning Objectives Catalogue Patient Safety into the existing undergraduate medical education [2], [3], [4]. Longitudinal curricula have the advantage that teaching contents are conveyed multiple times on increasing levels of difficulty and with growing complexity as well as various focus areas (see Figure 1 (Fig. 1)). For the topic of patient safety this means it can be taught within the context of the various clinical subjects and the canon of all medical disciplines. Knowledge on, as well as skills in and conducive attitudes towards patient safety will be acquired step-by-step in accordance with the students’ level of knowledge and experience. 

To implement a topic longitudinally, the curriculum has to be developed systematically and strategically orientated [3]. Even though the longitudinal integration of the GMA-LZK in the medical curriculum is the favoured goal, it can be constructive to initially proceed gradually (see Figure 1 (Fig. 1)).

For the Learning Objectives Catalogue Patient Safety to formally be considered complete, all learning objectives should be covered by mandatory courses. The weighting and allocation of the learning objectives for patient safety should follow the percentages for the various thematic blocks indicated in the learning objectives catalogue. Within the various thematic blocks discretionary decisions between quality and quantity will be necessary: For example it is an unanswered question whether a complete implementation of the GMA-LZK should be sacrificed in order to ensure a more didactic implementation of a chosen learning objective. As a rule of thumb: The more learning objectives are being implemented, the more likely the consolidation of the desired attitude towards patient safety, the greater the need, however, for coordination between the various teaching units [5]. However, it is important that completing a learning objective should not be confused with a on-off presentation of teaching contents.

Thinking ahead, not only the transparency of the learning objectives and the teaching contents should be considered from an early stage, but also their evaluation and examination. Therefore, each learning objective in the GMA-LZK has been concretised in regard to the three competence levels of knowledge, skills and/or attitude. This allows a competence-orientated examination of the learning objectives that have been achieved. An example for a specific learning objective solely orientated towards the competence level knowledge would be: "The student knows the differences between "guilt" and "liability" in the context of clinical action and can thus name them" (Learning Objective 1b in the GMA-LZK) [1]. Such knowledge-based skills, for instance, could be examined by means of Multiple Choice (MC) Tests. A learning objective orientated towards all three competence levels – attitude, knowledge and skills – could, for example, be on patient identification: "The student knows the principles of patient identification to be the active process of cognition, recognition and making certain. The student knows various methods for identifying patients and can apply those." (Learning Objective 3e in the GMA-LZK) [1]. The examination of such a learning objective could be included as a smaller section into a more extensive simulation scenario for the OSCE-Exam (Objective Structured Clinical Examination). Learning objectives focusing on attitudes pose a special challenge in regard to their examination. They can be assessed over a longer period of time by means of questionnaires, for example, measuring the attitudes and needs of medical students concerning patient safety [6].

## Where do we stand?

It is important to determine what kind of prerequisites and parameters regarding the topic of patient safety a faculty provides for, before making changes to a curriculum or initiating focused and constructive advancements. A well-executed situation analysis can ensure that

- a suitable connecting point to the curriculum and the faculty can be found as well as
- measures will be coordinated with the appropriate decision makers and that the implementation of those measures will last.

For determining the current situation at the faculty, we have drawn up questions that will support self-reflection of the current embedding of the topic of patient safety in the respective curricula (see Table 1 (Tab. 1)). With the help of the answers, a curriculum development can be structured and prepared. These questions, however, should not simply be processed one after another. They influence and complement each other making consulting them twice worthwhile.

Furthermore, already implemented courses on the topic of patient safety should be incorporated constructively in the overall curriculum to avoid doubling and to ensure that the teaching contents are coordinated appropriately in regard to their levels of difficulty and complexity. The so-called "curriculum mapping", which is used not least to determine a successful curriculum development, is an important aid and offers guidance when developing a curriculum.

Curriculum mapping was developed in the 1990s to integrate curricular contents and the respective examinations [7], [8]. In its present form, curriculum mapping is used primarily to identify unnecessary doubling, inconsistencies, weaknesses and gaps in a syllabus. The goal is to create maximal transparency of the study objectives, contents and examinations for lecturers and students, to – so to say – highlight the common theme of the patient safety curriculum, as teaching contents can only be coordinated thematically and didactically in a constructive manner when they are transparent. Curriculum mapping has become increasingly popular, since the method seems instinctively plausible and trivial. Behind this simplicity hidden are, however, a multitude of methods, templates and resources for the professional development of teaching institutions [8]. 

In regard to the topic of patient safety, we recommend using the GMA-LZK as the base for determining objectives or rather as a minimal standard guide for curriculum mapping. Questions that should be asked within the scope of a curriculum mapping are listed in Table 2 (Tab. 2).

Changes to a curriculum always pose questions regarding coordination. Therefore, the curriculum mapping should at least clarify whether there is any kind of steering coordination of the various courses and how strong its influence might be. Here, amongst other factors, it has to be considered whether a course is taught by a single lecturer or whether this course is embedded in a wider teaching concept of a discipline (e. g. internal medicine).

## Where can and do we want to go?

This next section explores realistic, feasible and verifiable objectives for embedding the topic of patient safety in a curriculum by looking at three "Best-Practice-Examples". The three examples illustrate how approaches of equal quality can differ depending on the parameters of the faculties – without one approach being better or worse than another one. Merely the strategies and short-term objectives on the path to an implementation vary.

### Freiburg – the "service provider" variation

The Albert-Ludwigs Universität Freiburg (University of Freiburg) coordinates the basic orientation and arrangement of the teaching courses for the undergraduate medical education centrally. The individual departments, however, are given great freedom in the actual selection and implementation of educational content, which makes the implementation of new subject areas – such as patient safety – comparatively uncomplicated. The topic of patient safety was first integrally introduced to in the winter term 2014/2015: Reorganising the cross section area 3 (health economics, health care system and public health care sector) made implementing essential objectives on patient safety possible. Ever since then, fundamental knowledge on the topics of teamwork and error management have been conveyed by means of the computer-based tool "eLearning Patientensicherheit" (ELPAS; eLearning patient safety).

For a lasting impact, however, as well as for the development of specific competences in the field of patient safety, additional courses are necessary. In the spirit of an "internal service provider" a team from the fields of medical psychology and medical sociology cooperates closely with the lecturers of the various subject areas to develop and coordinate the learning modules further. In regard to content, the sensitisation for the topic as well as specific knowledge development are paramount for a longitudinal development of the ELPAS. The selection of contents is influenced decisively by the learning objectives of the GMA-LZK. While the basic eLearning course (ELPAS), which is already available, offers an overview of the topics of teamwork and error management and ensures a discipline-independent embedding of these topics in the cross section area 3, the following in-depth modules within (the scope of) the Project ELPAS Longitudinal (ELPAS-L) focus specifically on particular aspects of patient safety in various medical specialist disciplines. Looking for example at the surgery and orthopaedist course, this means that an ELPAS-L module dealing specifically with the WHO–checklist for safe surgeries [http://www.who.int/patientsafety/safesurgery/checklist/en/] is being developed. In the paediatrics course, on the other hand, the emphasis is put on the communication with parents and children as well as the proper calculation of medication dosage. In doing so, the principles of taking actions in the interest of patient safety are integrated into a disciplinary context allowing for the knowledge to eventually transfer smoothly into practice and demonstrating the applicability of the principles. In addition, embedding the topic in subjects with a close contact to patients increases the significance of patient safety in undergraduate medical education. 

By embedding the topic of patient safety longitudinally and into specific contexts in the second section of studies, this topic becomes a "constant companion" throughout the entire course of studies. Following the "low dosage, high frequency approach" [9] the students are offered short study modules on patient safety within central clinical teaching course. These modules implement the learning objectives of the GMA-LZK using disciplinary examples while consistently building upon the prior knowledge of the students. That way, each semester the lecturers are teaching the topic of patient safety from a new perspective. A uniform logo for the topic facilitates recognition.

#### Bonn – the cooperative variation of implementation

The undergraduate medical education at the University of Bonn (Rheinische Friedrich-Wilhelms-Universität Bonn) is characterised by a decentralised coordination and structuring of the teaching courses. An important disciplinary resource for the topic patient safety is the Institute for Patient Safety (IfPS) with a professorial chair for patient safety. For other interdisciplinary teaching contents, such as professional communication, progressively centrally coordinated working groups are in the process of being formed. The goal in Bonn is to gradually embed the GMA-LZK longitudinally into undergraduate medical education while ensuring that the learning objectives of the GMA-LZK are conveyed in appropriated stages. Due to the decentralised coordination, a collaborative implementation strategy is being followed: When possible the collaboration takes place with other work and project groups so that the IfPS can integrate the topics of patient safety step-by-step into the existing teaching courses as soon as the responsible teaching coordinator shows interest or the teaching courses are being redesigned. That way, single teaching units on patient safety have already been implemented in two cross section blocks, namely the practical training for paediatrics and the practical year. The subjects of occupational safety, hygiene, patient safety and law are special in regard to their integration [10]. Those four subjects are taught within a teaching course at the beginning of each study section (pre-clinic, clinic, practical year). The teaching contents have been coordinated to match the chosen thematic areas with increasing complexity and feature practical relevance corresponding to each study section, so that at the start of the clinical study section, for example, the students are taught legal and hygienic aspects of blood sampling taking occupational and patient safety into consideration (Learning Objective 3e in the GMA-LZK, patient identification) [1].

#### Munich – the longitudinal complete implementation variation

The Office of Student Affairs at the Ludwig-Maximilians-University in Munich already has experience in curriculum development due to having implemented a longitudinal curriculum on the topic of communication as well as a longitudinal curriculum on scientific skills. With the support of the Office of Student Affairs and the allocation of personnel resources, work has started on initiating a complete implementation of a patient safety curriculum. For this purpose, two decisive preparatory steps have been taken: All existing courses that meet the learning objectives on patient safety have been identified. Moreover, a supervisory group that has dedicated itself to the implementation and is sufficiently visible within the faculty has requested support for the introduction of the curriculum "MeCuM Patientensicherheit" (Medical Curriculum Munich – Patient Safety) from the faculty council. During this waiting period the identified courses are being modified, supplemented or rearranged in accordance with the learning objectives. Furthermore, a "Corporate Design" is in the making, so that the introduction of the patient safety curriculum can be perceived, not only by the "insiders" (members of the faculty) but also by the "outsiders" (students, other universities), as a success that should be upheld. In the course of the curriculum development mentioned above, new courses will be implemented and reviewed. With the help of the supervisory group the responsibility for these courses will be distributed to the designated subject areas, so that they can last despite potential changes, for example in the personnel. Next to these tasks the supervisory group, the evaluation officers and the student body of the faculty in Munich cooperate closely to gather feedback from students on how the various courses have been implemented and on the coherence of the overall curriculum. When aiming for a longitudinal complete implementation there is, on the one hand, the danger of the "fragmentation of learning objectives", which means a lack of coherence between the various units and the overarching topic patient safety. On the other hand, the complete implementation offers the opportunity to review all courses "at once". Moreover, positive dynamics in the faculty can quickly lead to a great visibility for the students and lecturers as well as a significant learning progress for the students.

## How do we get there?

When aiming to sustainably embed a curriculum for teaching the topic of patient safety in the respective faculties, a relevant part of the organisation has to be changed as well: This means that every curriculum development is at the same time a faculty or rather an organisation development [2]. The continuous application of methods from the project management cycle can support this process. Useful questions from the project management are presented in Table 3 (Tab. 3).

### Requirements for lecturers on the topic of patient safety

International literature names various requirements that have to be met by lecturers teaching patient safety. These should also be taken into consideration when developing a curriculum: Lecturers should enable future doctors to apply safety-related skills in consideration of the respective context [11] and to develop leadership skills to independently initiate processes of change [12]. As per the WHO, lecturers should make use of the entire range of teaching approaches and methods [5]. Therefore, we recommend supplementing the GMA-LZK with the curriculum-guide of WHO (2009) [5], particularly for lecturers in the area of patient safety. This guide presents comprehensive teaching material in the form of didactic tools, methods as well as examination material for the content design of teaching units. Several of those teaching methods require particular teaching competences, such as technical and creative competences for designing simulation or blended-learning modules [13] and social as well as personal competences ensure, for example, successful debriefing sessions after simulations [14]. As the initial training, but also the continuing and advanced training in the field of patient safety ideally follows a multiprofessional approach, lecturers should at best be able to offer inter-professional teaching competences [14], [15], [16]. 

Patient safety is and will always be a dynamic field of knowledge. With increasing research on patient safety, new topics and competences will be established [17], that will then again have to be integrated into the local curricula and all learning objective catalogues. Not only lecturers but also curriculum planers have to be prepared for these developments.

## Conclusion

We have shown that curriculum planers can choose from various strategies and many connecting factors for systematically embedding the topic of patient safety in the curriculum and managing it sustainably. The questions presented in this article can help to determine the current situation of one’s faculty and make it possible to estimate how much personnel and time resources will be needed. There are established techniques that planners and planning groups can refer to for purposefully organising and monitoring a curriculum development as well as for making it visible.

As a committee we offer curriculum planers networking opportunities to discuss not only content and disciplinary questions, but also curriculum development and the embedding of the topic patient safety as well as the culture of patient safety in the medical curriculum. We see such networking as a chance to utilise synergies and to learn from and with each other in the context of a continuous improvement process.

## Note

The position paper was accepted by the GMA executive board on 20-09-2017.

## Competing interests

The authors declare that they have no competing interests. 

## Figures and Tables

**Table 1:**
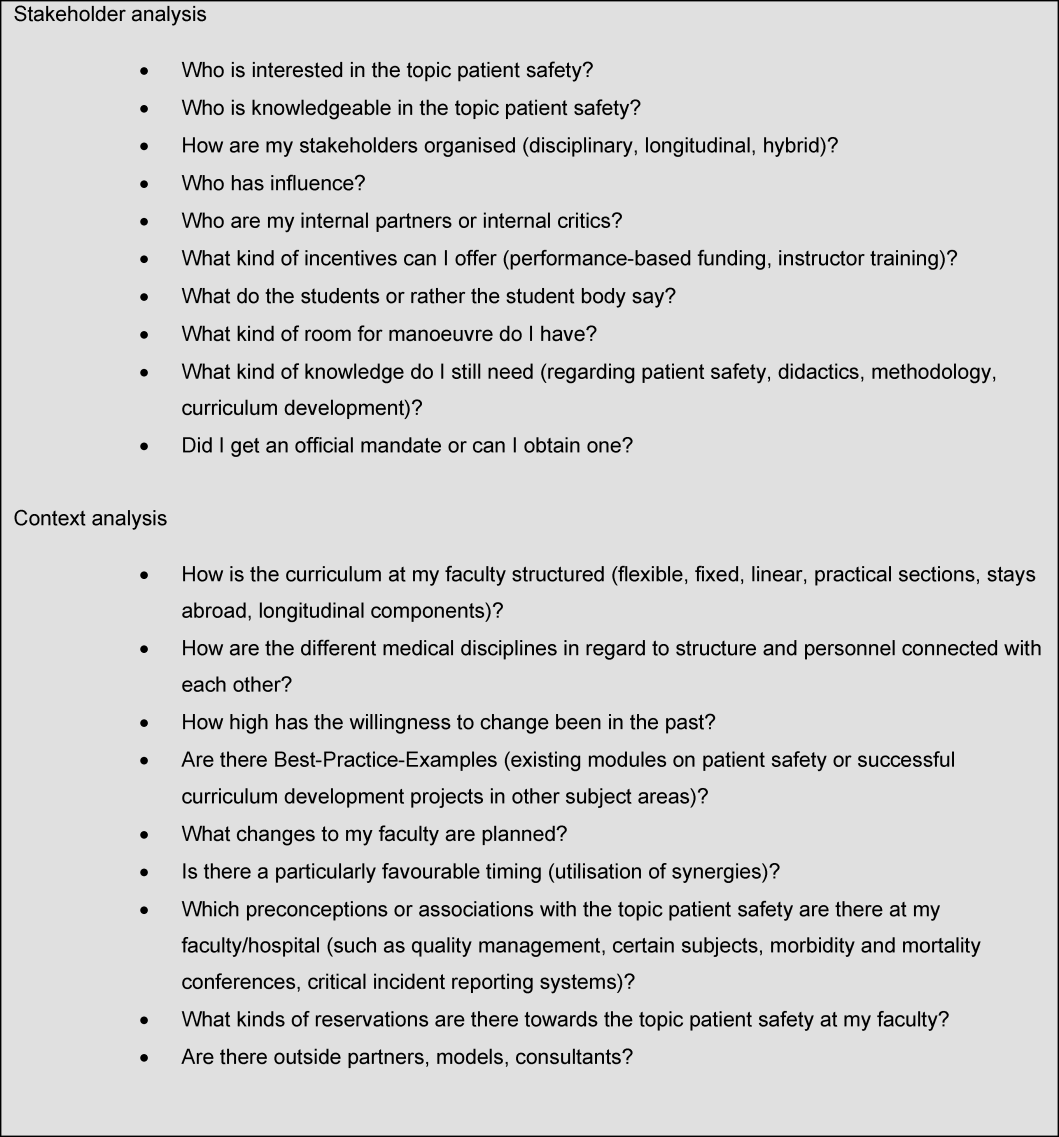
Questions for a structured situation analysis

**Table 2:**
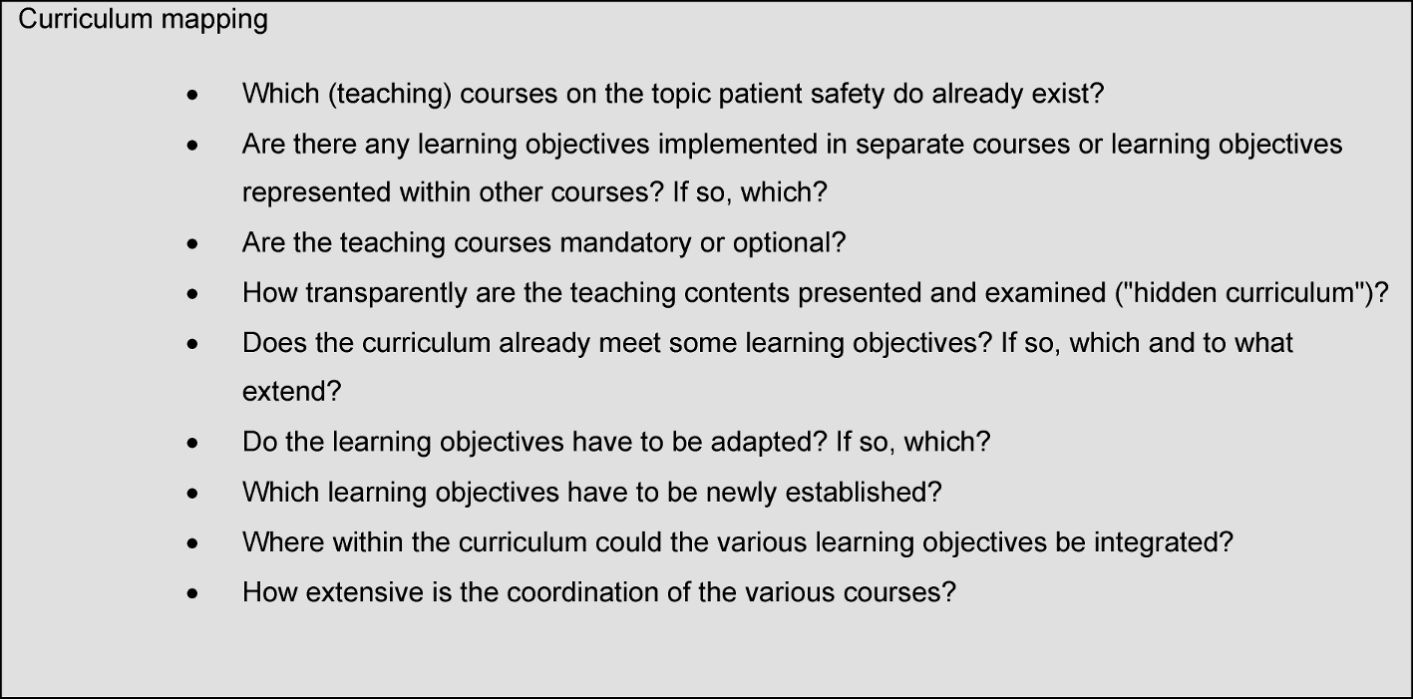
Model questions for a curriculum mapping

**Table 3:**
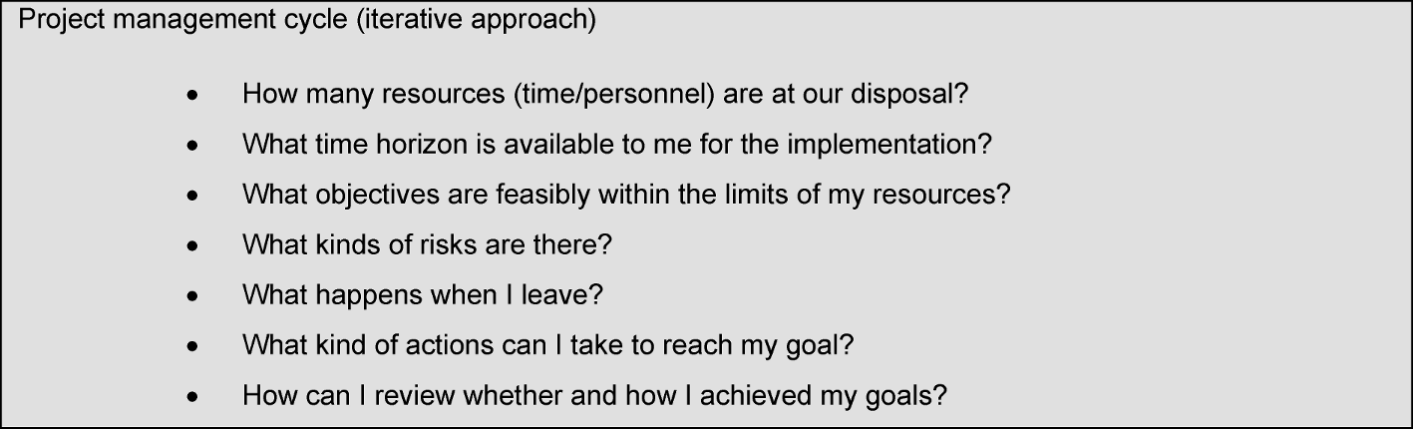
Selected questions for project planning the curriculum development

## References

[1] Kiesewetter J, Gutmann J, Drossard S, Gurrea Salas D, Prodinger W, Mc Dermott F, Urban B, Staender S, Baschnegger H, Hoffmann G, Hübsch G, Scholz C, Meier A, Wegscheider M, Hoffmann N, Ohlenbusch-Harke T, Keil S, Schirlo C, Kühne-Eversmann L, Heitzmann N, Busemann A, Koechel A, Manser T, Welbergen L, Kiesewetter I (2016). The Learning Objective Catalogue for Patient Safety in Undergraduate Medical Education—A Position Statement of the Committee for Patient Safety and Error Management of the German Association for Medical Education. GMS J Med Educ.

[2] Thomas PA, Kern DE, Hughes MT, Chen BY (2016). Curriculum development for medical education: A six-step approach.

[3] Sator M, Jünger J (2015). Von der Insellösung zum Longitudinalen Kommunikationscurriculum—Entwicklung und Implementierung am Beispiel der Medizinischen Fakultät Heidelberg. Psychother Psychosom Med Psychol.

[4] Silverman J (2009). Teaching clinical communication: a mainstream activity or just a minority sport?. Patient Educ Couns.

[5] WHO (2009). Who Patient Safety: Curriculum Guide for Medical Schools.

[6] Kiesewetter J, Kager M, Fischer MR, Kiesewetter I (2017). Validation of a German short version of the Attitudes towards Patient Safety Questionnaire (G-APSQshort) for the measurement of undergraduate medical students' attitudes to and needs for patient safety. GMS J Med Educ.

[7] Jacobs HH (1991). Planning for curriculum integration. Educ Leadership.

[8] Jacobs HH, Johnson A (2009). The curriculum mapping planner: Templates, tools, and resources for effective professional development.

[9] Sutton RM, Niles D, Meaney PA, Aplenc R, French B, Abella BS, Lengetti EL, Berg RA, Helfaer MA, Nadkarni V (2011). Low-dose, high-frequency CPR training improves skill retention of in-hospital pediatric providers. Pediatrics.

[10] Steudel H, Stümpfig A, Manser T, Rösing C, Engelhart S, Münster E (2016). ArHyPa–Best: Arbeitsschutz-Hygiene-Patientensicherheit als Basiskompetenz bester Mediziner -innovatives Lehrprojekt an der Medizinischen Fakultät der Universität Bonn. http://dx.doi.org/10.3205/16gma187.

[11] Jones AC, Shipman SA, Ogrinc G (2015). Key characteristics of successful quality improvement curricula in physician education: a realist review. BMJ Qual Saf.

[12] Ahmed M, Arora S, Baker P, Hayden J, Vincent C, Sevdalis N (2013). Building capacity and capability for patient safety education: a train-the-trainers programme for senior doctors. BMJ Qual Saf.

[13] Henriksen K, Dayton E (2006). Issues in the design of training for quality and safety. Qual Saf Health Care.

[14] Kolbe M, Weiss M, Grote G, Knauth A, Dambach M, Spahn DR, Grande B (2013). TeamGAINS: a tool for structured debriefings for simulation-based team trainings. BMJ Qual Saf.

[15] Walkenhorst U, Mahler C, Aistleithner R, Hahn EG, Kaap-Fröhlich S, Karstens S, Reiber K, Stock-Schröer B, Sottas B (2015). Position statement GMA Committee—"Interprofessional Education for the Health Care Professions". GMS Z Med Ausbid.

[16] Aktionsbündnis Patientensicherheit e. V. (2014). Wege zur Patientensicherheit Lernzielkatalog für Kompetenzen in der Patientensicherheit.

[17] Long S, Arora S, Moorthy K, Sevdalis N, Vincent C (2011). Qualities and attributes of a safe practitioner: Identification of safety skills in healthcare. BMJ Qual Saf.

